# BioBridge Prepares Students to Successfully Bridge into High School Biology Through Cancer Research Education

**DOI:** 10.15695/jstem/v8i1.01

**Published:** 2025-01

**Authors:** Michelle S. Johnson, Kathy C. Haynie, Lalita A. Shevde, J. Michael Wyss

**Affiliations:** 1 Center for Community Outreach Development (CORD), The University of Alabama at Birmingham, Birmingham, AL; 2 Haynie Research and Evaluation, Skillman, NJ; 3 O’Neal Comprehensive Cancer Center and Department of Pathology, University of Alabama at Birmingham, Birmingham, AL

**Keywords:** Cancer, Youth Enjoy Science, STEM Education, Biology, Bridge to High School, Informal Summer Education

## Abstract

A lack of academic and social readiness limits the successful transition to high school and college for many students from populations historically underrepresented in Science, Technology, Engineering, and Math (STEM) and historically underrepresented groups in biomedical careers (HUG). Also, many families from underrepresented groups share little family health information, leading to suboptimal early health awareness, testing, and treatment. BioBridge is a 5-day summer learning program offering interactive experiences to facilitate successful transition into high school biology and to introduce students to cancer biology and biomedical careers. It also helps students gain important insights and skills and be effective communicators who can encourage discussions about cancer biology and family health history, potentially increasing early detection and treatment of cancer and decreasing its adverse effects. Over the past three summers, 97 students have participated in BioBridge and significantly increased their knowledge of biology, cancer, and cancer-related careers. They have also begun to understand the connections between science and social and ethical issues. Participants used a video platform to create discussions about cancer biology, which they then shared with their families. They found that BioBridge boosted their willingness and confidence to discuss cancer and, subsequently, other health topics with their families.

## INTRODUCTION

Cancer is the second leading cause of death in both Alabama (Alabama Statewide Cancer Registry, 2021) and the US (Browne, 2023). Although significant progress has been made in the prevention, detection, and treatment of cancer, elevated rates of breast (Crown and Joseph, 2022, prostate (Lillard, 2022), and lung cancer (Zavala et al., 2021) exist in medically underserved populations. Moreover, while overall cancer rates are falling in Alabama and the US in both majority and minority populations, the incidence rates and overall mortality rates continue to rise in Black females under the age of 50 (National Cancer Institute, 2024). Further, the 2016–2020 age-adjusted incidence rates for colon and rectal cancer in Alabamians (compared with all US residents) were over 12% higher, and lung cancer rates were over 11% higher (National Cancer Institute, 2024). In 2023, Alabama had an estimated 30,730 new cancer cases, including prostate (5,320), female breast (4,500), lung and bronchus (4,280), colon and rectal (2,570) cancer, and melanoma (1,510) (Harris, 2023; Siegel et al., 2023). Moreover, in both females and males, Blacks (compared to Whites) have higher cancer incidence and mortality rates (Harris, 2023). These patterns contribute to limited early testing and treatment, inadequate access to care, and educational inequalities (Brady et al., 2022; Chaudhary, 2022; Ponce et al., 2022).

### The Importance of Educating Students About Cancer.

Students’ cancer knowledge can directly impact their family members’ health. Disproportionate levels of wealth, socio-economic status, employment benefits, and education contribute to disparities in cancer mortality rates. About 40% of all cancers in the US are caused by modifiable risk factors (Williams et al., 2023). Thus, BioBridge provides middle school students with cancer education and awareness, helping them to understand modifiable risk factors and encouraging early testing and detection for them and their families. Activities that clearly reduce the incidence, morbidity, and mortality of cancer include early detection via cancer screening, awareness of potential inherited risks, lifestyle changes, recognition of the influence of health disparities, research, innovations in the treatment of patients, and support for caregivers, and communication of health issues within families (Copeland et al., 2023; Daniel et al., 2021; Tinker et al., 2019). As the importance of early cancer detection and treatment becomes increasingly evident, it is crucial for teenagers to engage in conversations about cancer.

Disparities in cancer outcomes have been linked to a lack of diversity in the oncology workforce (Crown and Joseph, 2022; Ponce et al., 2022). Further, medical mistrust and perceived discrimination also contribute to worsened health outcomes in women, Blacks, and Hispanics (Bazargan et al., 2021). In part, this results from disparities in education and engagement, which leads to divergent biomedical careers. According to the National Science Foundation, Blacks or African Americans, Hispanics or Latinos, American Indians or Alaska Natives, Native Hawaiians, and other Pacific Islanders are underrepresented in nearly all STEM or health-related careers (National Science Foundation, 2023). Initiatives such as the National Cancer Institute’s (NCI) Youth Enjoy Science (YES) Research Education grant program can create a sense of belonging for students from underrepresented backgrounds, thus increasing their engagement, preparedness, entry, academic success, and retention in diverse cancer-related careers (Chaudhary, 2022; Lin et al., 2022; Patel et al., 2021; Rookwood et al., 2022).

Introducing students to STEM at a formative age can demystify science, increase a sense of belonging, and build excitement about STEM subjects. The University of Alabama at Birmingham’s (UAB) Center for Community Outreach Development (CORD), in partnership with UAB’s O’Neal Comprehensive Cancer Center, developed BioBridge, a one-week inquiry-based STEM education program for rising 9th-grade students. BioBridge introduces students to cancer biology research, cancer awareness and screening, and STEM careers (specifically cancer-related careers). BioBridge was designed to serve as a bridge from middle to high school, especially in preparing students for high school biology. BioBridge was founded on CORD’s Summer Science Institute (SSI), which for more than 25 years has provided high school students with engaging opportunities to participate in STEM learning. CORD also offers comprehensive summer and school-year STEM learning opportunities for students ranging from grades 3 through 12 (Chandran et al., 2020; Dizbay-Onat and Wyss, 2018; Jarrett et al., 2016; Patel et al., 2021).

### Informal Learning Education.

A significant portion of learning occurs through informal education experiences and STEM summer camps, which can contribute to students’ understanding of science, encourage classroom STEM education and discussions, and introduce STEM careers (Eshach, 2007). Informal STEM learning environments offer educational activities outside of traditional classrooms and are often called extra-curricular activities, non-formal education, and co-curricular activities (Gross and Rutland, 2017). For example, these include programs in natural history museums, science centers, zoos, botanical gardens, aquariums, nature centers, and community-based centers (Adams, 2020). Informal learning is defined as “the sum of activities that comprise the time individuals are not in the formal classroom in the presence of a teacher” (Gerber et al., 2001). Out-of-school educational resources, such as informal educational programs, increase STEM learning in children, e.g., by increasing problem-solving, creativity, interest, and understanding of complex topics (Falk and Meier, 2021; National Research Council (NRC), 2015). Further, informal STEM learning environments encourage curiosity and exploration through discovery learning (Lindeman, 2020), enhance middle school students’ STEM interest and engagement through interactive experiences such as hands-on activities (Christensen et al., 2018) and provide an environment in which learning exploration is nurtured (Worsley, 2022).

This manuscript describes the development, implementation, and outcomes of Central Alabama’s first cancer biology-focused summer learning opportunity. The curriculum was designed to better equip students for high school biology, enhance their scientific literacy, and foster positive attitudes toward science, research, and STEM careers. Additionally, it introduces students to various STEM career paths and the significance of cancer biology research within those fields. Results from the initial three years of the program reveal that students acquired knowledge and shifted their perspectives regarding STEM careers following their participation in BioBridge. Moreover, BioBridge prompted students to initiate open discussions about health, cancer, disease prevention, and treatment strategies within their families.

## METHODS

The goal of the summer program was to 1) enhance foundational knowledge and create engagement in cancer biology 2) create a pipeline of students from underrepresented backgrounds who are excited to enter STEM majors, like those offered UAB’s Undergraduate Cancer Biology major; and 3) increase interest and understanding of cancer among students’ family members and the community.

This study used the National Institute of Health’s (NIH) definition of individuals from underrepresented racial or ethnic groups (e.g., Black or African American, Hispanic or Latino, American Indian or Alaska Native, Native Hawaiian or Pacific Islander), individuals with disabilities, individuals from rural areas, disadvantaged backgrounds, and women (National Institute of Health, 2023) as being underrepresented in health-related sciences.

### Recruitment.

BioBridge recruited rising high school freshmen from the metro Birmingham area for six sessions (2 sessions per year) in the summers of 2021–2023. BioBridge recruited students through 1) CORD’s previous middle school camps participants, 2) recommendations from 8th-grade teachers in area school systems, 3) web-based recruiting through CORD’s website, 4) presentations at area churches and schools, 5) student recommendations from past BioBridge participants, and 6) an educators’ symposium held at the O’Neal Comprehensive Cancer Center. Prospective students were required to submit application packets consisting of 1) a recommendation from a science teacher describing a student’s interest and aptitude, 2) a recent school transcript, and 3) a signed statement of commitment to attend all days of the camp. A total of 97 rising 9th-grade students completed BioBridge during the summers of 2021, 2022, and 2023. The participant group included students from 29 different schools in the Greater Birmingham Metropolitan area.

### Student Facilitators Were an Essential Element in BioBridge.

UAB undergraduate “YES” research scholarship recipients were selected to be facilitators/mentors based on their 1) willingness to be near-peer mentors, 2) prior research laboratory experience, 3) previous mentoring experience, 4) availability to attend all training sessions, and 5) communication and leadership skills.

The undergraduate student facilitator role was an essential part of the BioBridge model. Prior to the first BioBridge session, student facilitators underwent a one-week training program to familiarize themselves with the specific STEM teaching experiences they will provide, the related educational kits (see Appendix for specific kit details and ordering information), and the program activities. They were selected based on their expertise in particular areas and their STEM majors to assist in developing and leading specific BioBridge activities.

Examples include (1) Cancer Biology majors developing and guiding cancer tumor modeling activities and (2) Genetics and Genomics Sciences majors leading the DNA structure and function activities.

At the end of each day of BioBridge, student facilitators met with the program director as a group. They reflected on daily icebreakers, modeling activities, cancer biology-based games, lecture content, and the effectiveness of invited UAB faculty speakers and facilitators.

### Curriculum Development.

Two to three months before the start of BioBridge, the UAB “YES” scholarship recipients (undergraduate students) attended instructional meetings either virtually or in person. These meetings involved developing a cancer biology curriculum that was aligned with Next Generation Science Standards, HS-LS1, HS-LS3, and HS-LS4 (Lee et al., 2014; Pruitt, 2014), and the Alabama Course of Study in Science (2015) Standards, 1a, 3a, 3b, 3c, 4, 11a, and 11c (Bice, 2015).

BioBridge was created to tackle the challenges of engaging students in effective, inquiry-based biology learning experiences. The following design principles were incorporated into the pedagogical framework of BioBridge to create an engaging experience: 1) BioBridge emphasized the use of hands-on activities (Kyere, 2017), e.g., cell staining, and it provided real-world contexts linking STEM concepts to everyday life (Dare et al., 2021) e.g., BioBridge participants learned the science behind UV-induced DNA damage and how it could lead to skin cancer. BioBridge also utilized experts across several fields to interact with the students. Pathologists, graduate students, genetic counselors, etc., provided activities and encouraged meaningful discussions. For instance, the students explored UV-induced DNA damage and methods to decrease the adverse effects of UV exposure to the skin. To facilitate the learning, social interactions and collaborations (Ellwood and Abrams, 2018; Pasani and Amelia, 2023) were encouraged as BioBridge participants performed group activities, encouraging them to work together as a team, share ideas, and solve problems collaboratively. BioBridge also integrated the use of technology like DNA gels and digital microscopes to enhance participants’ learning (Yang and Baldwin, 2020). Using these principles, BioBridge worked to bridge academic gaps, enhance biomedical knowledge, and foster meaningful, hands-on learning experiences for students transitioning from middle school to high school biology.

### Content Knowledge Assessment.

A 20-item biology content knowledge assessment was developed for participants to complete as a pre/post measure at the beginning and end of their BioBridge camp experiences. The initial assessment, comprising multiple-choice questions, was matched to 9th-grade biology concepts, basic cancer awareness knowledge, and questions related to the healthcare needs of the medically underserved. The biology content knowledge assessment was piloted in Year 1 (summer 2021) and found unreliable in terms of alignment to BioBridge content. Given the content and construct validity evidence, the assessment was revised for subsequent BioBridge programs. Items on the pilot biology content knowledge assessment were subsequently either retained, revised for content and clarity, or replaced in the final version of the assessment, utilized as a pre/post assessment with BioBridge 2022 and 2023.

### Knowledge, Interest, and Self-Perceptions Survey.

During BioBridge’s first year, a survey was developed and validated to assess participants’ knowledge, career interests, and self-perceptions. The questions on the survey focused on key concepts in BioBridge and are shown in Table 1. Content validity was achieved by adapting items like those of other validated STEM interest surveys, such as the S-STEM (Mahoney, 2010), including validation of items by subject matter experts.

Participants were asked to rate their current levels (immediately before the BioBridge experience) and their levels after participating in BioBridge using a 5-point Likert scale (1=low, 5=high). The survey was piloted with BioBridge participants at the end of the BioBridge camp in the summer of 2021. Based on 24 participant responses to the 12 survey items, the value for Cronbach’s Alpha was α = 0.8014. This served as evidence of good internal consistency for this 12-item survey (e.g., construct validity) and added to the validity evidence for using this survey with future BioBridge students.

### Student Evaluation of The Overall Camp Experience.

At the end of BioBridge 2021–2023, participants provided an overall quality rating (five-point scale, 1=poor to 5=excellent) for their BioBridge experience. They were also given a series of open-ended questions to provide feedback on their camp experience. The questions were:

1. What was your favorite part of the BioBridge program? Why?
2. In the past week, did you have conversations about cancer biology with anyone outside of the BioBridge Camp? If so, please describe your conversation.
3. Is there anything else you would like to tell us?

### Survey Data.

Camp participants were given open-ended surveys at the end of each week of camp to determine the impact of participation in BioBridge (see Methods section). Eighty-six percent of BioBridge participants voluntarily completed the post-camp survey. Data were combined (n=83) across the six BioBridge sessions (two cohorts per year: 2021 [n=16; 8], 2022 [n=11; 16], and 2023 [n=16; 16]).

### Statistical Analysis.

The analysis methods for the biology content knowledge assessment were carefully planned at both the item and test levels. First, all valid participant responses from the summers of 2022 and 2023 were compiled. Next, these responses were scored. At the item level, the percent correct (P+) was calculated for each item by dividing the number of correct responses by the total number of responses. Student’s t-tests were then conducted to determine if there were significant gains or losses in P+ scores from pre- to post-assessment. Items responses were analyzed to identify any potential issues, such as low-performing response options, particularly focusing on easy items (P+ > 95%), difficult items (P+ < 25%), and items with no change in scores from pre- to post-assessment.

At the test level, Cronbach’s alpha was calculated for both the pre-assessment and post-assessment to measure internal consistency, with a score of .60 or higher deemed acceptable for a low-stakes test aimed at evaluating a single construct. Finally, a plot of pre- and post-assessment P+ scores was analyzed to ensure that nearly all items fell above the identity line and that a range of item difficulties was represented in both assessments (data not shown).

For the Likert scale items, statistical analysis was performed using one-way ANOVA and Tukey’s post hoc analysis. Items were considered significant with a p-value of < 0.05.

### Institutional Review Board.

This research study was approved by the University of Alabama Institutional Review Board as an exempt program for educational assessment. All surveys were completed anonymously and submitted voluntarily.

## IMPLEMENTATION

### BioBridge Curriculum.

**Figure 1.**
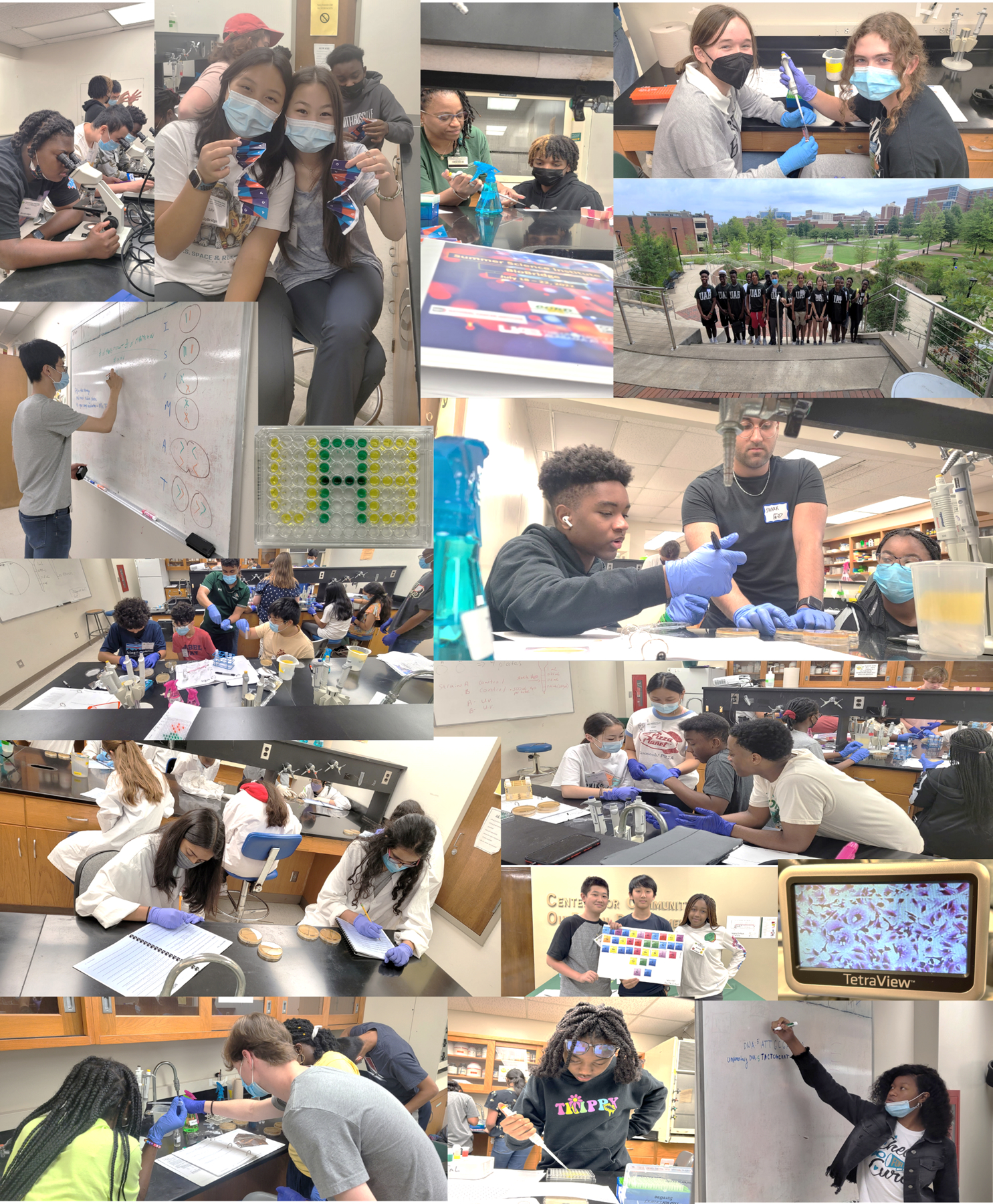
Selected Photos Showing Camp Activities. Representative photographs of activities performed during a typical BioBridge session. See supplementary methods for a breakdown of each day’s structure with kits and vendor information.

The rising 9th graders participated in hands-on, inquiry-based, scientific exploration and learning daily during the week-long BioBridge camp. At the beginning of the week, students were assigned to groups of three to five for all activities/experiments. Group assignments remained the same for the duration of the week. Daily sessions began at 9 a.m. with an icebreaker to develop social familiarity (camp participants and undergraduate student facilitators). They concluded at 3 p.m. each day for five-day sessions and at 4 p.m. for four-day sessions (these were held during the 4th of July week, and thus the day of the 4th was excluded. The camp director, undergraduate students, or guest speakers offered short PowerPoint lectures (less than 10 minutes), followed by a related lab (see Figure 1).

#### Day 1.

The camp director gave a lab safety overview, presented the learning objectives, considered what the students would gain from the experience, and explained participant expectations for the week. After the overview, all students took part in an initial learning activity - an introductory lecture on topics that included cell cycle, mitosis, and cancer - delivered by a UAB Cancer Biology undergraduate student. At the conclusion of day one, camp participants engaged in an overview of modeling tumor development from normal tissue. Participants were asked to share their models at home and discuss cancer development and progression with friends and family members. Some participants recorded videos of this at-home activity (and received “extra” team points). The ability of assigned teams to earn points was a deliberate strategy to foster motivation and teamwork within groups. The groups (by earning points through daily challenges such as reviews and daily quizzes) encouraged the camp participants to engage and collaborate with each other while fostering friendly competition with other groups. Moreover, camp participants worked collectively for group and individual goals.

#### Day 2.

A daily icebreaker was followed by a review of day one content through Kahoot, a game-based platform that can be used in the place of traditional quizzes (Cutri et al., 2016, June; Wang and Tahir, 2020). Campers were then introduced to proper pipetting techniques via video and printed resources. Micropipetting training followed. In the afternoon, campers reviewed the structure and function of DNA, and each participant created a DNA origami model (https://www.genome.gov/about-genomics/teaching-tools/dna-origami). After modeling DNA, campers reviewed genes and hereditary traits leading to mutations and cancer.

During the summer, BioBridge partnered with the UAB Medical Scientist Training Program (MSTP) students who were actively engaged in cancer research to create an activity that fostered scientific thinking, problem-solving, and investigation. MSTP students led an activity with this focus, followed by a discussion on careers in research and medicine. At the conclusion of the day, campers were given instructions to take their DNA models home and discuss the composition of a DNA molecule, i.e., nucleotide bases, phosphate backbone, sugar molecules, and hydrogen bonding. Again, participants who shared recorded videos were given “extra” team points.

#### Day 3.

Following an icebreaker, Day 3 activities focused on UV-induced cancers, specifically melanoma. Campers were introduced to the concept of UV radiation and learned about how the DNA in their skin cells can be affected by UV radiation, the type of mutations caused by UV damage, and the importance of using sunscreen to reduce the risk of skin damage and skin cancer. Thereafter, the students conducted a hands-on activity using a UV-sensitive yeast strain that campers first grew and tested. Various modes of sun damage prevention were examined, including sunscreen and thin pieces of cloth; aluminum foil was used as a control. After the plates were dried, the yeasts were exposed to UV radiation, and students semi-quantified the results. This hands-on approach provided an excellent opportunity for campers to generate hypotheses, discuss the scientific method, and the experimental variables, and interpretation of the data. As in the previous day, CORD partnered with UAB’s Department of Pathology’s Residents’ Outreach program. During the residents’ visits, campers were invited to use microscopes to observe the architectural differences between various human tissues from different organs (including normal and cancer tissue). The residents concluded their visit by providing information on pathology careers such as anatomic pathology, surgical pathology, and laboratory medicine. The day concluded with a Kahoot review and instructions for subsequent at-home video discussions.

#### Day 4.

A lecture on current cancer detection methods and therapies followed the morning icebreaker. The associated activity focused on cancer detection using biomarkers and blood antigens. As a mock clinical specimen, simulated blood samples were used to detect human papillomavirus (HPV) and prostate serum antigen (PSA). Next, campers evaluated the yeast plate growth that started on Day 3. Visual analysis of the yeast plates fostered conversations about anticipated versus actual results and experimental variables, leading to new hypotheses. Each camper was asked to generate a testable hypothesis based on their new experimental results. Later, the campers were introduced to cancer genetics and genetic counseling as a career. The campers next acted in the role of “genetic counselors” who were investigating sporadic, familial, and hereditary causes of cancers using genetic testing and pedigree analysis. The day concluded with the campers presenting their weeklong group project, followed by a cancer biology-based Jeopardy game covering all BioBridge activities and lectures.

#### Day 5.

The final day of camp was “Fun Friday”. It involved a university campus tour, including classroom buildings, student housing, the recreation center, the student center, and a state-of-the-art research area. During the tour, campers were given information about UAB STEM majors and STEM career opportunities, including careers not traditionally considered STEM. Campers were given information on preparing for college life and success. At the student center, campers separated into groups and participated in a scavenger hunt to solve puzzles using knowledge from all the BioBridge learning modules. Following the scavenger hunt, the final presentation of the week was given by a Ph.D. graduate student studying the effects of drug targeting in glioblastoma. The student concluded by describing how her undergraduate research helped prepare her for a career as a cancer biologist.

Following the 5-day STEM experience, campers were asked to complete the post-camp survey to assess gains in cancer biology knowledge, career exposure, research exposure, and self-identity. Qualitative and quantitative data from pre- and post-test surveys were submitted to Haynie Research and Evaluation for analysis (McGill et al., 2019).

## RESULTS

### Demographics.

BioBridge was created and implemented to assist rising 9th-grade students in successfully bridging into high school biology and introducing them to cancer biology. During the summers of 2021, 2022, and 2023, 97 students have participated in BioBridge. There were no significant differences in gender, racial, or ethnic diversity among the 6 cohorts of student participant cohorts. Black and Latinx students accounted for 41.8% of student participants (Table 2). Data were not collected on preferred pronouns, disability, economic or rural status.

### Content Knowledge Results.

**Figure 2.**
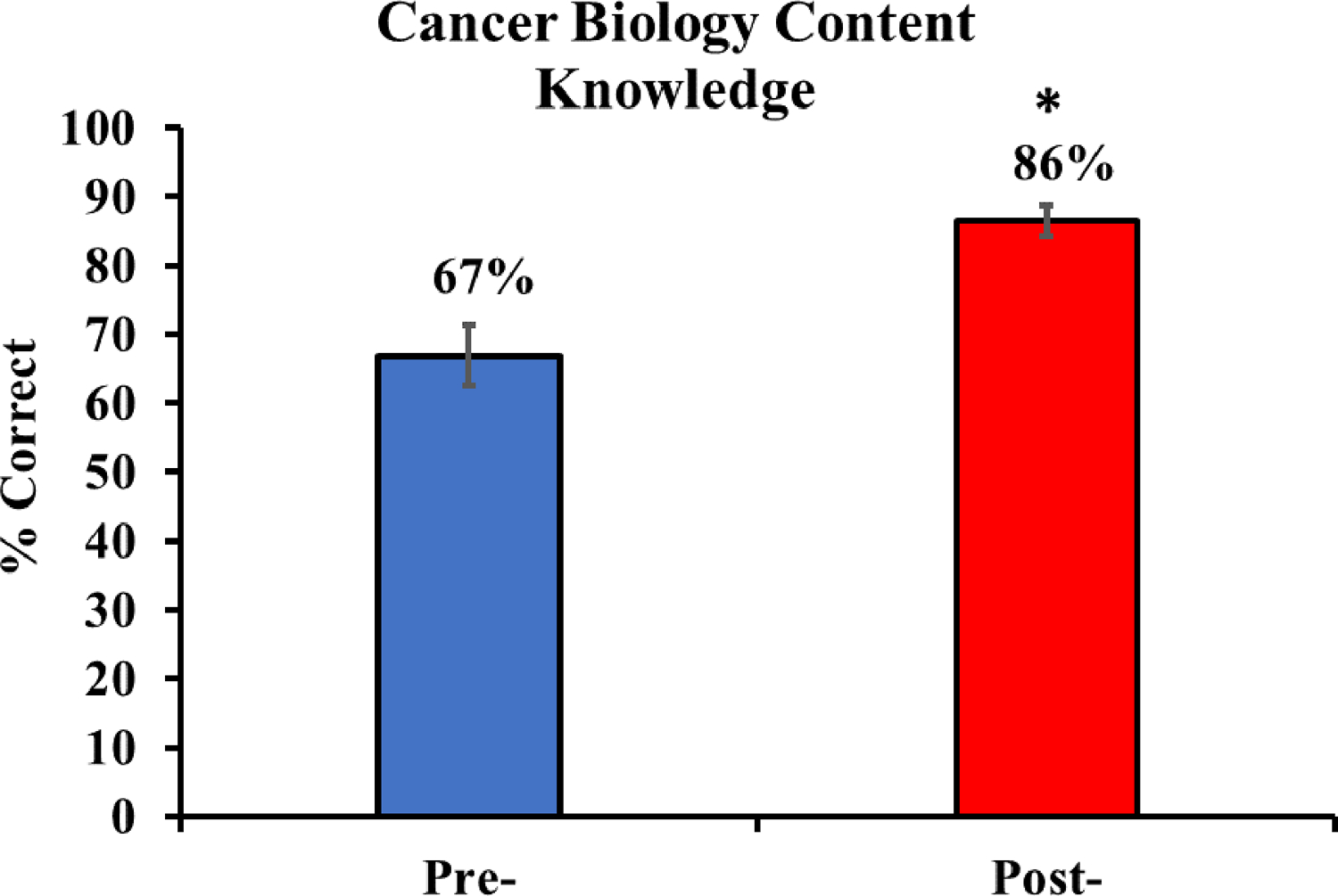
Cancer Biology Content Knowledge. Figure 2 provides evidence that students’ content knowledge increased significantly from before to after BioBridge. In the summer of Years 2 (2022) and 3 (2023), rising 9th-grade students in both BioBridge sessions completed a 20-item content knowledge assessment at the beginning and end of their BioBridge camp experiences. Data represent means ± s.e.m., n = 68. The graph shows the combined results for 68 students (students present on the first and last day of camp) across the two years (between-year differences were non-significant). * p = 0.001 compared to pre-testing. Statistical analysis was performed using One-way ANOVA.

In the summer of years 2 (2022) and 3 (2023), the students’ content knowledge increased significantly from pre- and post-BioBridge, as measured by the BioBridge biology content knowledge assessment (Figure 2). Year 1 (2021) data also showed a significant increase in pre- to post-BioBridge test scores (from 68% to 85%; Student’s t-test, p < 0.001). However, for years 2–3, content knowledge questions were updated based on the year 1 testing and to align with the revised curriculum and learning activities, and thus, the year 1 data was not combined with the year 2–3 data. Questions about medically underserved populations in Alabama were also removed from year 2–3 testing since these topics are not covered in year 2–3 BioBridge offerings. There were no significant differences between the years 2022 and 2023 testing. We note that duplicate response items and items with fewer than six responses were excluded from the analyses. The remaining 68 responses were analyzed.

The biology content knowledge data exhibited good reliability both pre (α = 0.772) and post (α = 0.742). The items exhibited an appropriate range of difficulty for all groups. On the pre-assessment, the percent correct (P+) for the 20 items ranged from 37% to 97%, with an average of 67% correct. The two items with P+ > 95% were retained due to the unique content they assessed. On the post-assessment, P+ values ranged from 68% to 99%, with an average of 86% correct and an average pre-post increase of 19% across the 20 items. For 19 of the 20 items, P+ values increased; one item (the only true/false item) exhibited a small decrease. All the other items were multiple-choice items. Test-level analyses of pre-and post-P+ values indicated that 95% of items were above the identity line (potentially demonstrating knowledge gains). Based on t-test comparisons, 65% of the items showed significant pre- to post-BioBridge gains; initial P+ values for four items were very high. Question 19 (central dogma) increased from 36.8% to 86.8% correct. Further, questions 2 (cell division and cancer), 6 (lung cancer mortality), 9 (characteristics of metastasis), and 20 (nitrogenous base found in RNA) all showed P+ gains of over 30%. Individual assessment questions and performances are documented in Supplementary Figure 1 and Supplementary Table 1. These findings highlight the effectiveness of the assessment in capturing meaningful learning gains and suggest that the BioBridge biology content knowledge assessment is a reliable tool for measuring knowledge improvements over time.

### Students’ Knowledge, Interest, and Self-Perceptions.

**Figure 3.**
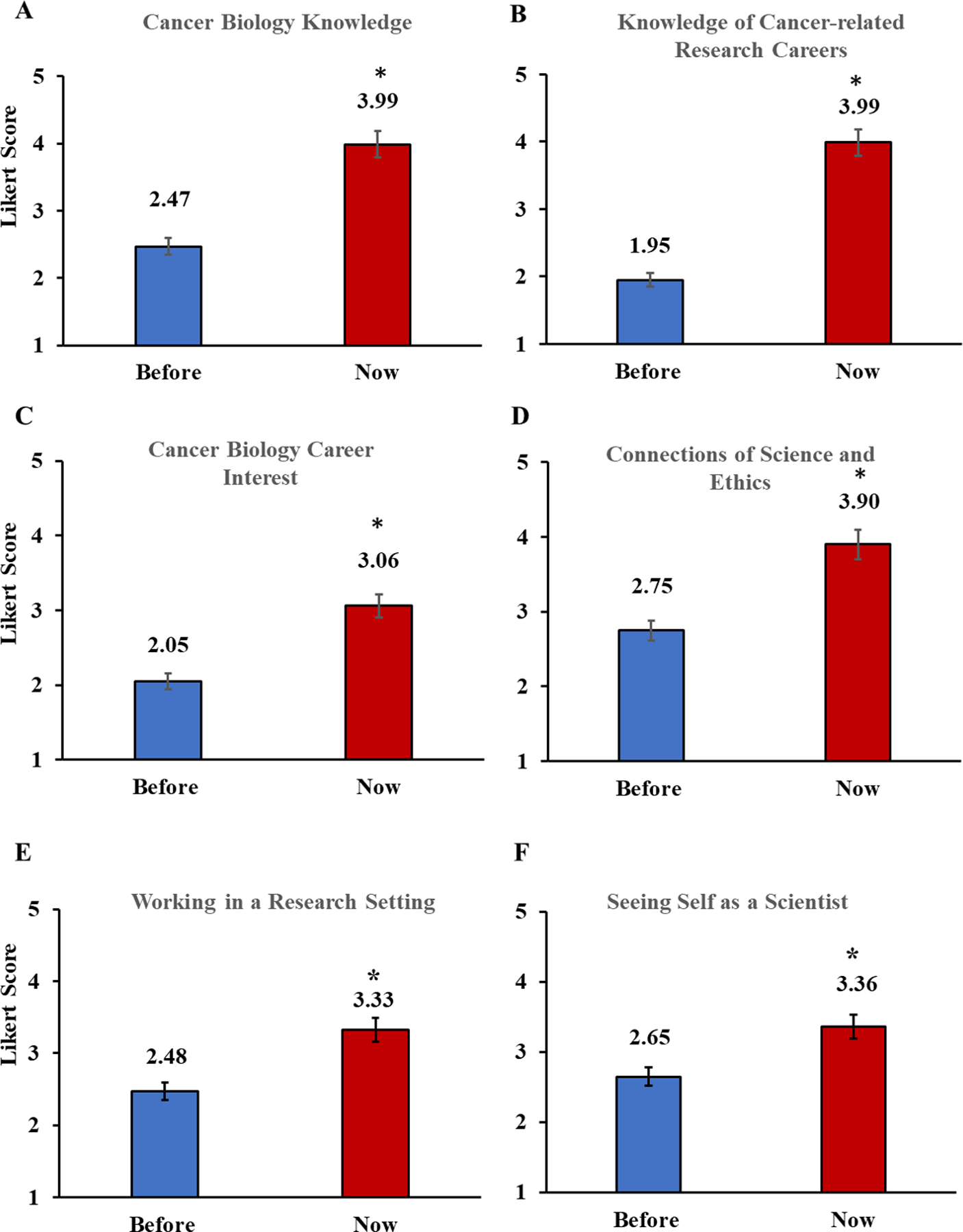
Students’ Knowledge, Interest, and Self-perceptions. Figure 3A–F shows average Likert scores for student perceptions (1=low to 5=high). The student’s knowledge of cancer biology and cancer research careers increased significantly from before to after participation in the BioBridge program. These data are combined (n=83) across six BioBridge sessions: session 1 2021 (n=16), session 2 2021 (n=8), session 1 2022 (n=11), session 2 2022 (n=16), session 1 2023 (n=16) and session 2 2023 (n=16). Data represent means ± s.e.m., n = 83. * p < 0.05 compared to before BioBridge. Statistical analysis was performed using One-way ANOVA and Tukey’s Multiple Comparisons test.

Figure 3A–F indicates that the participants gained significant knowledge of cancer biology and cancer research careers from the BioBridge program and self-perceptions and understanding of social and ethical issues. There were no significant differences among the sessions; therefore, these data were combined across six BioBridge sessions (n=83); note that these items were the same for all six sessions; 2021–2023). Cronbach Alpha analysis was performed to determine the reliability of the self-perception survey (with incomplete responses removed, n-77). The Cronbach Alpha for the summers of 2021, 2022, and 2033 were α = 0.8014 (n=24), 0.7864 (n=26), and 0.9324 (n=27), respectively. Across all summers combined, the alpha was a= 0.8687 (n = 77). Statistical analysis demonstrated significant gains in all six areas:

- Knowledge of cancer biology, +1.52, F(1, 164)=104.80, p=.000
- Knowledge of research careers, +2.04, F(1, 161)=220.76, p=.000
- Working in a cancer research-related career, +1.01, F(1, 161)=29.98, p=.000
- Science connected to social and ethical issues, +1.15, F(1, 162)=51.77, p=.000
- Seeing yourself as a scientist, +0.71, F(1, 164)=13.32, p<.001
- Working in the research setting, +0.85, F(1, 161)=16.11, p=.000

Based on the three-year dataset, student gains from BioBridge were robust. In combination, the findings provide strong evidence that BioBridge increases the students’ content knowledge, knowledge of and interest in cancer-related careers, understanding of the connections of science to social and ethical issues, and interest in working in a research setting.

### Student Ratings of their BioBridge Experience.

**Figure 4.**
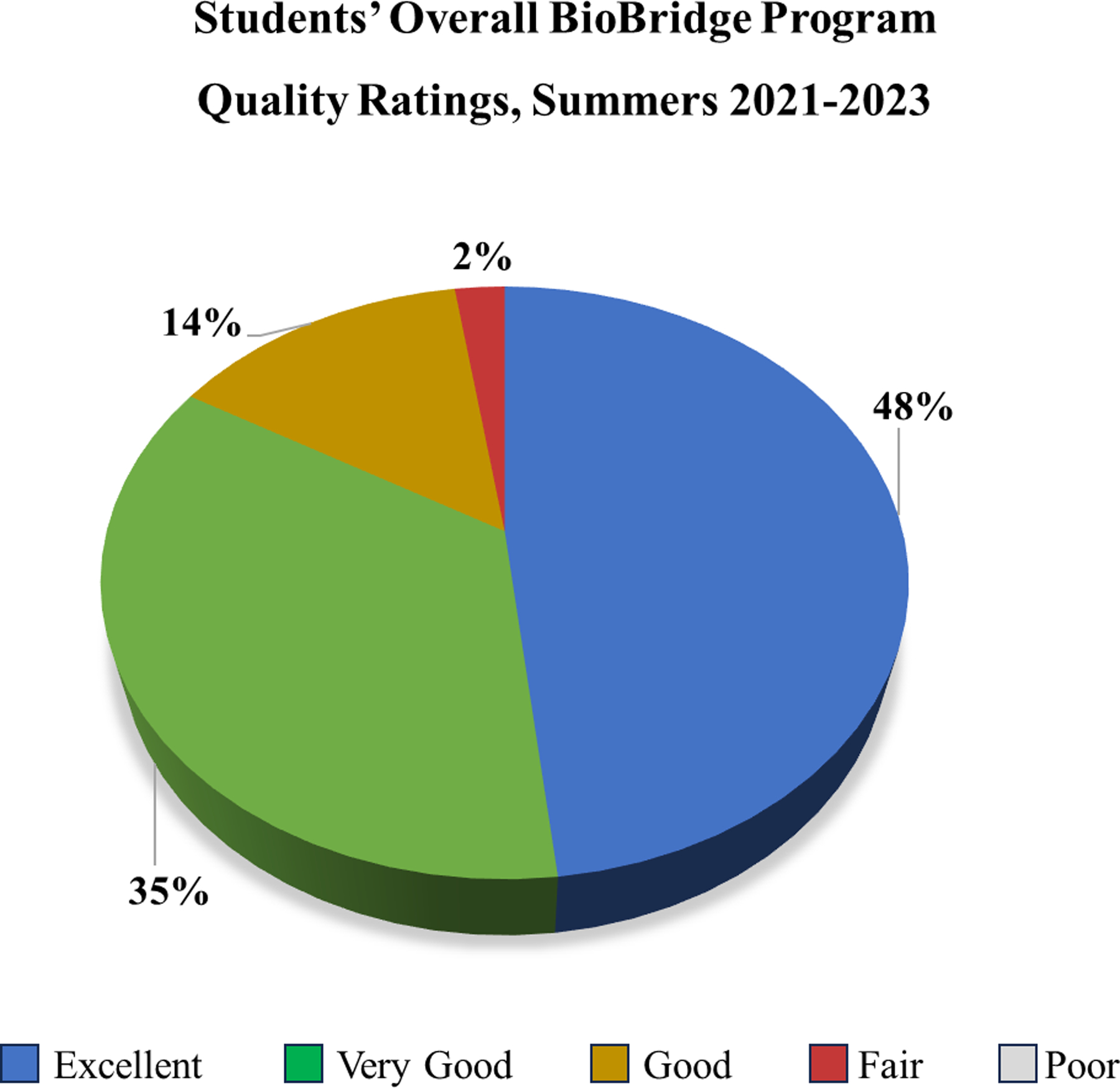
Overall BioBridge Program Quality Ratings. Figure 4 provides the overall quality ratings of BioBridge from students in Years 1, 2, and 3, n=83. Nearly 84% of the students in the years 2021, 2022, and 2023 gave BioBridge an overall rating of very good or excellent.

The students gave BioBridge high overall quality ratings for the summers of 2021–2023 (n=83), with 84% of the rising 9th-grade participants across all three summers giving BioBridge an overall rating of very good to excellent (Figure 4). No significant differences were found based on racial or sex self-identifications.

Campers were also given a series of open-ended questions to provide feedback on their camp experience. One student reported their favorite part: “*My favorite part was the fun experiments we did even though we were learning at the same time.*” Another student reported that BioBridge stimulated their career interest, “*I had a conversation with a Cancer Biology Graduate Student about how she got past rough parts and why she chose what she does, and it helped me make up my mind on what I want to pursue.*”

Assisting students to successfully bridge from middle school to high school, especially in biology and other STEM areas, was a primary goal of BioBridge. It also led to an opportunity to share cancer knowledge in the greater community. As shown in Supplementary Table 2, camper reflections indicated that students liked BioBridge and wanted to share more cancer knowledge with their family and friends, and this stimulated biomedical career interests in many of the students.

### Community Engagement.

**Figure 5.**
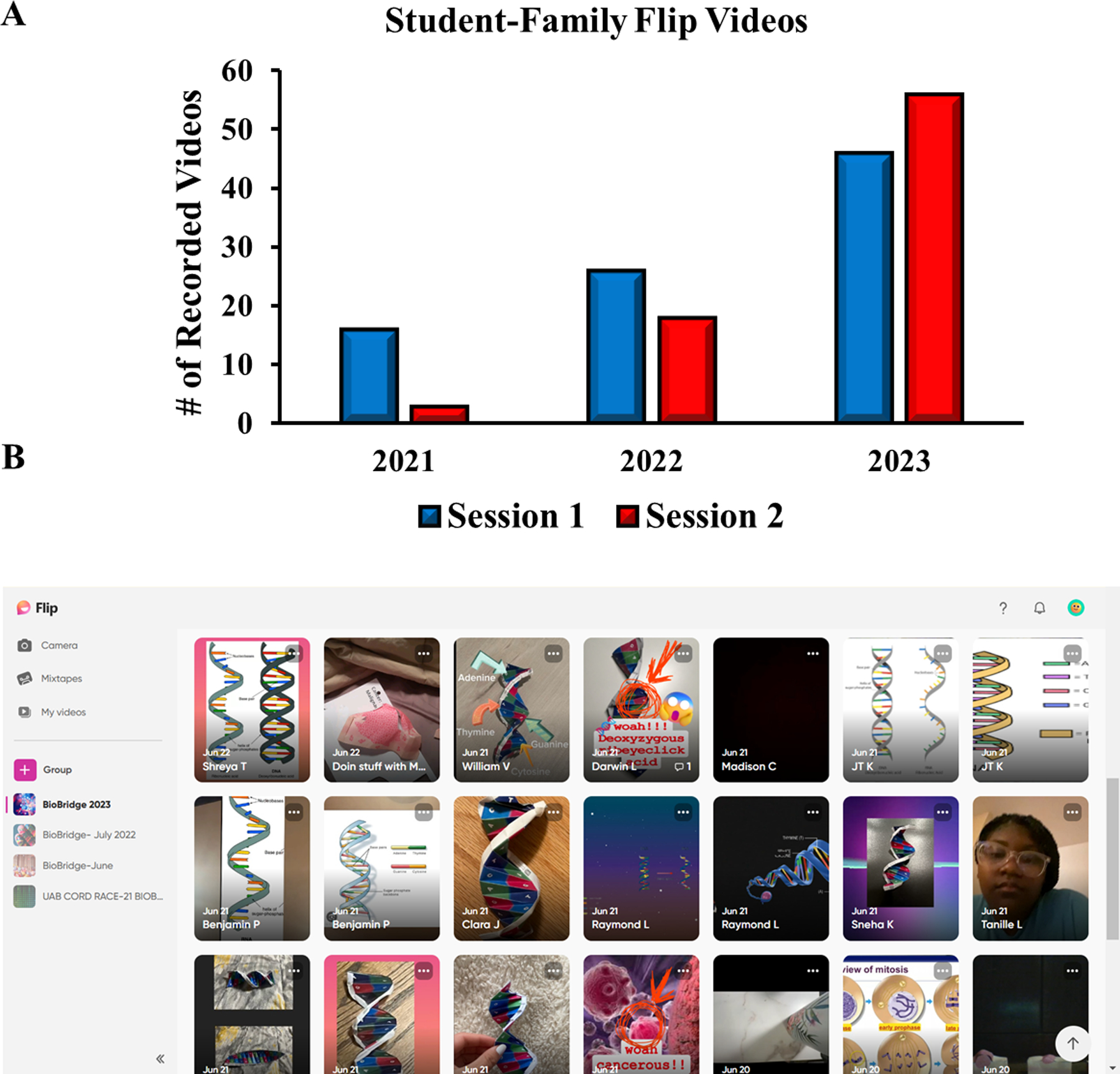
Student Flip Videos. Panel A shows the increase in Flip Video participant submissions over three summers. Videos increased from 19 videos in 2021 to 44 videos in 2022, to 104 video responses in 2023. Panel B represents a screen capture of submitted video responses on the Flip website.

One of the primary objectives of BioBridge was to enhance engagement and communication with the broader community regarding the learning gains of BioBridge students, as well as their awareness and understanding of cancer and diseases. Teaching in the 21st Century requires the use of readily available technology to enhance student learning and sharing of experiences (Figure 1). BioBridge used Flip (formally FlipGrid) as an effective video platform to facilitate social learning and student interaction with family members and the public (McClure and McAndrews, 2016; Stoszkowski, 2018; White, 2015). At the end of each day, camp participants were instructed to create a video of discussions that they would have with their parents, friends, siblings, or other family members related to what they had learned about cancer and biomedical research that day. For example, on day 1 (2022), students created a video detailing the steps involved in mitosis, which they shared with either a parent or other relatives/friends present. In 2023, students were given the option to model and discuss the development of a malignant tumor from normal tissue using Origami Organelles. Several strengths have been identified using this type of approach. For example, a QR code or invitation link is shared with students, and they can access the application through a cell phone, computer, or tablet and do not need to create an account for use. Positive reinforcement in the form of points awarded to “teams” was used to increase student participation, engaging their competitive team spirit. As shown in Figure 5, video responses increased from 19 videos in 2021 (37% of participants) to 104 video responses in 2023 (78%). Secondly, the percentage of students participating in video responses increased from 37% in 2021 to 78% in 2023 (data not shown). Further, “students shared cancer knowledge with their family and friends,” as shown in selected student reflections in Supplementary Table 2.

## DISCUSSION

BioBridge is an inquiry-based 4- or 5-day summer learning program offering interactive and collaborative experiences to increase the ability of students to successfully transition into high school, especially relative to biology and other STEM subjects, and to introduce them to cancer biology and biomedical research and careers. BioBridge is the first-of-its-kind program in the Birmingham metro area. It is one of a few National Cancer Institute Youth Enjoy Science R25 programs in the Southeast United States.

### Perceptions, Motivation, and Retention.

Many students lose interest in STEM subjects during the middle to high school transition period. Major contributing factors for this loss of interest include lack of academic readiness, experience with real-world science applications, role models in STEM, and overall fears surrounding STEM, e.g., the perception that science is too difficult (Coleman, 2018). These factors contribute prominently to the lack of inclusion of underrepresented populations in STEM education and careers (Carpi et al., 2017; Miriti, 2020). Informal science experiences like BioBridge have increased student interest and engagement in science, improved academic readiness in science, and introduced some STEM career opportunities to students. Several studies have reported that programs in anatomy (Heise et al., 2020), engineering (Asiabanpour et al., 2010), neuroscience (Chudler et al., 2018), physics (Dizbay-Onat and Wyss, 2018), and cancer research (Rookwood et al., 2022) are effective in lessening these disparities. Racial inequalities that exist in STEM are due to many factors, including lack of engaging middle and high school educational opportunities, lack of exposure to enrichment learning, consistent mentoring, and parental time constraints, resulting in a low percentage of students from underrepresented backgrounds (versus others) pursuing STEM majors and STEM careers. In 2021, the STEM workforce was composed of 15% Hispanic and 9% Black individuals. However, only 16% of people from underrepresented populations in STEM occupations had at least a bachelor’s degree. Additionally, minority populations collectively made up 31% of the overall population but only 24% of the STEM workforce. That same year, historically underrepresented minorities accounted for 26% of those obtaining a bachelor’s degree, 24% of those obtaining a master’s degree, and only 16% of those obtaining a terminal degree in STEM-related fields (“National Science Foundation,” 2023). These data highlight the need for opportunities to overcome such disparities.

BioBridge graduates reported increased knowledge and interest in cancer biology, improved knowledge of cancer-related research careers, and enhanced ability to see themselves as scientists working in a research setting (Figure 3). These gains were significant (p< 0.001) in all cases and robust across all six summer sessions and groups of BioBridge participants. Students’ education about cancer biology, hands-on lab experiences, exposure to role models (e.g., the UAB R25 Youth Enjoy Science (YES) scholarship recipients and the MSTP trainees), and participation in a learning community were all important aspects of the BioBridge experience.

These findings suggest that BioBridge and potentially other similar integrated educational programs can help deepen students’ knowledge and confidence in pursuing specialized scientific fields, impact students’ career aspirations, foster more well-rounded scientific literacy for students and can provide direct exposure to working in a research setting. Early and immersive experiences such as BioBridge can help foster future scientists and informed citizens (Talafian et al., 2019). These cumulative experiences led to gains in content knowledge and significantly more positive perceptions of the student’s potential for becoming future scientists. Thus, BioBridge has the potential to increase the participation of students from underrepresented populations in biomedical-related careers.

Disadvantaged populations often bear the brunt of health disparities (Schillinger, 2021). Family discussions on health and wellness are important for maintaining and improving health outcomes, especially regarding preventable diseases such as cancer. These conversations, for example, can help family members adopt healthy behaviors such as healthy eating, exercising, smoking cessation, and cancer screenings. By using Flip as an informal learning model, BioBridge focused on increasing community engagement through student, parent, and caregiver interactions. As shown in Figure 5, student-family engagements increased across multiple summers. During the one-week sessions, students’ Flip videos focused on the importance of seeing a genetic counselor, the benefits of using sun protection for melanoma prevention, cancer research pros and cons, and potential therapies etc. Unfortunately, the number of interactions before BioBridge was not measured and warrants further investigation. Based on unpublished observations, many students had few prior discussions about family health status with family members. Finally, the use of student-family interaction video recordings offered a unique platform by which the rising 9th graders were able to effectively communicate and potentially reach the community at large.

## LIMITATIONS AND LESSONS LEARNED

There were several limitations to this study. First, the initial content knowledge assessment was developed before the camp curriculum had been finalized. Several items were removed and/or optimized following the initial summer sessions in 2021. For example, a question regarding the percentage of the Alabama population that is medically underserved was removed from years 2 and 3 because this content was not covered during the one-week camp. Questions related to the biology content covered in the camp were added to the content knowledge assessment. The content assessment used in the summers of 2022 and 2023 reflects the more content-aligned version of BioBridge. Second, the effect of pre-testing on post-testing outcomes has been shown to enhance memory and retention, an effective pedagogical technique to enhance learning, especially when combined with exposure to new content (Latimier et al., 2019; Pan and Sana, 2021). It is possible that a test-retest effect had a negligible impact on score gains, given the short testing interval. The content areas with the most significant gain were aligned with the areas given the most focus during the BioBridge program (e.g., central dogma, cell division and cancer, lung cancer mortality, characteristics of metastasis, and the nitrogenous base found in RNA), suggesting that actual content learning impacted these gains. Third, Cronbach’s Alpha is commonly used in science education to measure the internal consistency or unidimensionality of the items within an assessment. These are acceptable internal consistency levels for low-stakes testing with a unidimensional test (Taber, 2018). However, given that the content knowledge assessment measures two concepts, biology content knowledge and cancer biology content knowledge, achieving unidimensionality was not the goal. The content knowledge assessment given to the BioBridge students can be considered multidimensional since the topics covered (9th-grade biology and cancer biology) are broader than the singular topic of high school biology. A subsequent factor analysis of the assessment data would provide evidence of whether a two-factor model (biology knowledge and cancer biology knowledge) fits the data.

Pre- and post-test instruments can be valuable tools for assessing the effectiveness of STEM camps such as BioBridge and are common in informal education programs (Herce-Palomares et al., 2022). However, it should be remembered that their quality and usefulness depend on many factors. Supplementary Table 1 summarizes the individual instruments, providing a starting point for preliminary stage assessment compilation, and can be amended based on curriculum content to determine student proficiency (pre-test) and knowledge gains (post-test).

Another limitation of this study was the lack of formal survey follow-up with these students to explore the long-lasting effects of the BioBridge training on content understanding of the graduates and the program’s long-term effects on increasing communications about disease within families or other associates. However, several students from BioBridge have participated in CORD’s Summer Science Institutes I and II (SSI-I and II) for rising 10th and 11th graders. And, when the course directors of those programs asked for feedback on their BioBridge experience from a summer or two ago, they offered the following testimonials:

*BioBridge was my second CORD Camp that I attended, and honestly one of the best summer science camps I’ve attended to this day…. Without BioBridge, I don’t believe I would’ve been able to foster this passion for learning, which I eventually plan to turn into a career in Oncology.* (DG, 2022)

*This camp was a cancer research-based intensive, and my teacher was Mrs. Michelle. She made me fall in love with science even more. We studied cancer cells and how mitosis occurs within the cell for an entire week.* (AH, 2021)

Furthermore, SSI- I and II students who participated in BioBridge were asked if BioBridge helped in their preparation for 9th-grade biology. While all students queried, responded “yes,” actual survey data was not quantified.

## CONCLUSIONS

BioBridge is an inquiry-based classroom and kinesthetic cancer biology experience intended to successfully transition students to high school in the fall. It is the first cancer biology-focused camp in Central Alabama, and its curriculum provides activities that engage students in biology, improve science literacy, and increase students’ overall positive attitudes toward science, research, and careers in biomedicine. Camp participation favorably impacted students’ knowledge about cancer biology and cancer-related research careers and their appreciation of biomedical ethics in its first three years. It also increased the participants’ interest in considering a career in cancer biology. The graduates and many of their parents considered that the program significantly increased their knowledge about biology and cancer and that this was an important aid for successful progression into and through high school. The program also helped the students communicate cancer knowledge to their families and appears to have opened many families up to talk about their health and familial health risk factors. At the end of the week, ~84% of the participants rated their BioBridge experience as very good to excellent. This is a significant finding in that the students gave up a week of summer vacation or sleeping in late to study hard-core biology.

The findings presented in this manuscript lay the foundation for implementing experiences like BioBridge. Moreover, the positive outcome measures of BioBridge provide an entrée to expanding camps of this nature to include all students. Finally, like other YES programs, BioBridge focused on active learning, near-peer mentoring, and early research associations, thus strengthening the students’ confidence that they belong in STEM and can effectively communicate their biomedical knowledge with their community and family.

### Reflections.

Reflecting on the implementation of BioBridge over the past three summers, a number of questions should be addressed. First, “What did not work in the BioBridge implementation and why?” In year 1 of BioBridge, participants gave relatively low ratings to the leadership team’s knowledge and tailoring of activities to learners’ needs. When participants were asked what they would change, their responses included more experiments, better preparation of supplies, better games, and more fun activities. Conversely, participants indicated their favorite activities were doing experiments and interacting with guest speakers (including near peers). Using this feedback, we shaped year 2 to include more hands-on activities/experiments, fewer and shorter PowerPoint presentations, games, and activities that were vetted prior to camp, and a requirement that all undergraduate facilitators had to attend a mandatory training session.

The students provided annual feedback to the question, “What worked very well?” By the end of year 3, all participants agreed that the facility was conducive to their learning and that the facilitators showed mastery of the subject matter. This was a large step forward from the year 1 participant feedback. BioBridge was further strengthened (in year 3) through undergraduate student facilitators employing their research knowledge. For example, undergraduate students majoring in Cancer Biology led the introduction to cancer. Students majoring in Genetics and Genomics Sciences led the lecture on DNA mutations, and students with extensive research skills were heavily involved in experimental preparation and activities.

As year 4 of the summer outreach approached, we asked, “What can we do next to improve learning and accessibility?” Despite BioBridge’s efforts to promote inclusivity, the number of underrepresented students has remained outside the target of 60%. Barriers such as lack of transportation and lack of parental engagement have limited this participation. This year, we have formed partnerships with local school systems, educators, the community, and STEM professionals to develop formal mentoring programs to increase the inclusion of all students and their parents (Bolshakova, 2019; Fadeyi et al., 2020). Finally, it will be important to conduct longitudinal assessments of BioBridge graduates to gain valuable insights as to the long-term impact of BioBridge on participants’ educational and career trajectories. We hope to use end-of-year data to probe participants’ academic performance (grades, test scores), STEM engagement (enrollment in advanced STEM courses and choosing a STEM major in college), college preparedness (entrance exam scores), and career aspirations (interest in pursuing STEM-related careers).

## Supplementary Material

Supplementary Figure 1

Supplementary Methods and Tables

ASSOCIATED CONTENT

Supplemental material mentioned in this manuscript can be found uploaded to the same webpage as this manuscript.

## Figures and Tables

**Table 1.** Knowledge, Interest, and Self-perceptions Survey.

Survey Questions		
	Before BioBridge	After BioBridge
**1**	My knowledge of the science of cancer biology BEFORE participating in BioBridge	My knowledge of the science of cancer biology NOW
**2**	My knowledge of different cancer-related research careers BEFORE participating in BioBridge	My knowledge of different cancer-related research careers NOW
**3**	My personal interest in working in a cancer research-related career BEFORE participating in BioBridge	My personal interest in working in a cancer research-related career NOW
**4**	My understanding of how science is connected to social and ethical issues BEFORE participating in BioBridge	My understanding of how science is connected to social and ethical issues NOW
**5**	Imagining myself working in a research setting BEFORE participating in BioBridge	Imagining myself working in a research setting NOW
**6**	Seeing myself as a scientist BEFORE participating in BioBridge	Seeing myself as a scientist NOW

Note: Students were asked to rate their knowledge of cancer biology and cancer-related research careers, interest in working in a cancer research-related career, understanding of connections to social and ethical issues on a 5-point Likert scale.

**Table 2.** BioBridge participant demographics over three summers of STEM camps.

	2021	2022	2023
**Male**	15 (56%)	15 (52%)	20 (49%)
**Female**	12 (44%)	14 (48%)	21 (51%)
**Black**	7 (26%)	14 (48%)	15 (37%)
**Latin X**	4 (15%)	0 (0%)	0 (0%)
**Asian**	11 (41%)	9 (31%)	23 (56%)
**White**	5 (19%)	6 (21%)	3 (7%)
**Total**	**27**	**29**	**41**

Note: Numbers in parentheses represent the percentage of the year’s student cohort. Over three summers, 97 students have participated in the cancer biology STEM camp.

## ABBREVIATIONS

CORD: Center for Community OutReach Development
HUG: Historically Underrepresented Groups
MSTP: Medical Scientist Training Program
NCI: National Cancer Institute
NIH: National Institute of Health
STEM: Science, Technology, Engineering, and Mathematics
SSI: Summer Science Institute
UAB: University of Alabama at Birmingham
YES: Youth Enjoy Science
