# Supplementary figures and images for: BioBridge Prepares Students to Successfully Bridge into High School Biology Through Cancer Research Education
Source: J STEM Outreach. Author manuscript; Available in PMC 2025 Sep 18. (PMC12442933; doi:10.15695/jstem/v8i1.01)

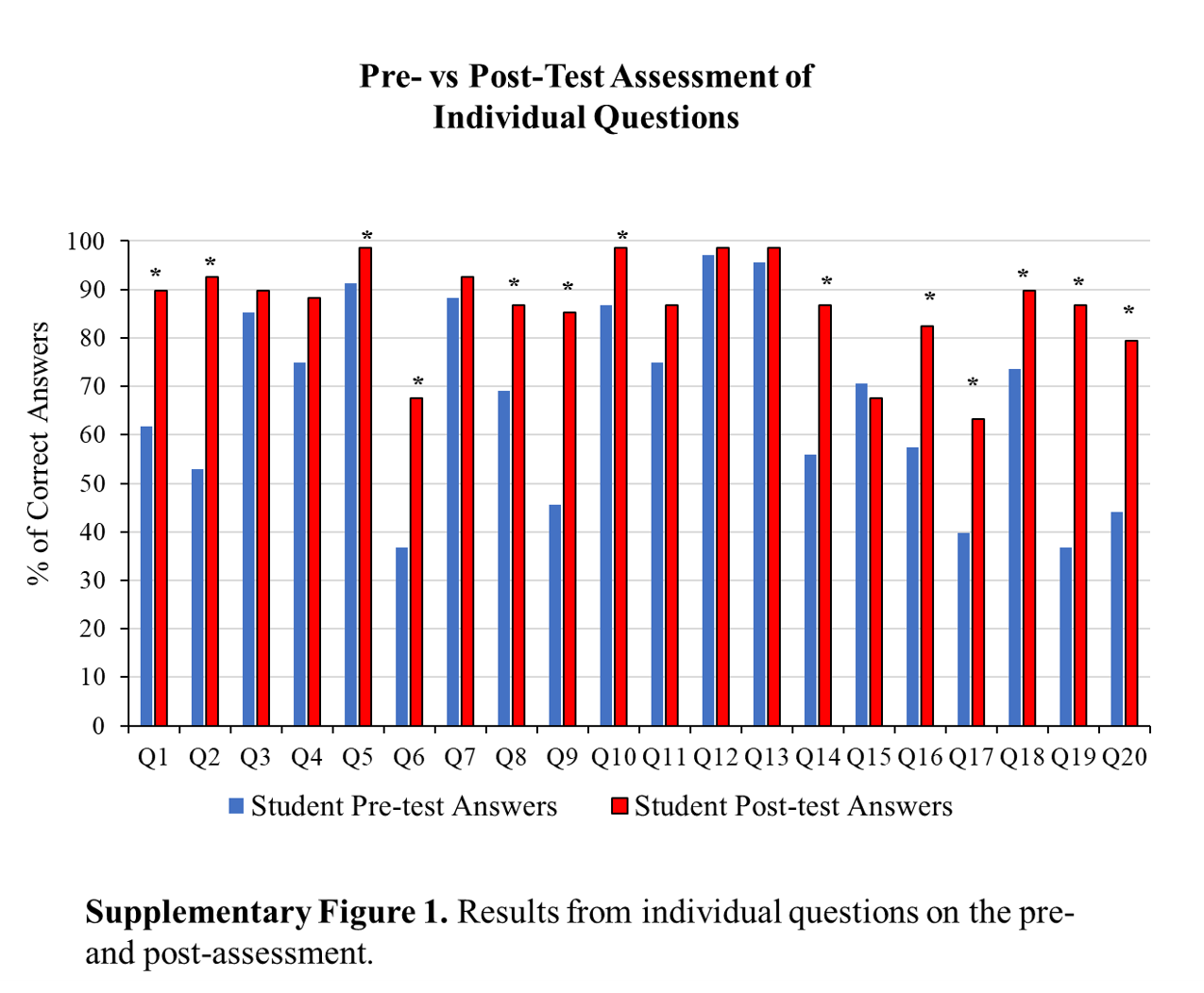

Supplement: Supplementary Figure 1 [file NIHMS2105949-supplement-Supplementary_Figure_1.png]
