# Supplementary material for: BioBridge Prepares Students to Successfully Bridge into High School Biology Through Cancer Research Education
Source: J STEM Outreach. Author manuscript; Available in PMC 2025 Sep 18. (PMC12442933; doi:10.15695/jstem/v8i1.01)
Supplement: Supplementary Methods and Tables [file NIHMS2105949-supplement-Supplementary_Methods_and_Tables.docx]

**SUPPLEMENTARY MATERIAL**

**Supplementary Methods**

**Daily Camp Structure:**

The general layout of the day 1 included:

- Introductions
- Lab Safety
- Icebreaker, group assignments, and team names
- The importance of cancer research
- What is mitosis: lesson, observing, and modeling
- How to use microscopes and analysis of mitosis using onion root slides (Carolina BioKits, item # 171130, Onion Mitosos)
- Morphology of Cancer Cells (Evotek, item # 990).
- Cancer origami (Edvotek, item # EVT-117)

The general layout of day two included:

- Daily icebreaker
- Review Kahoot
- Pipetting Activities (MiniPCR Micropipetting Activity, Item # KT 1510-10 and Bio-Rad, Microplate Art Pipetting: STEAM Activity, Bulletin# 6620)
- DNA structure and function (DNA Origami, National Human Genome Research Institute retrieved from: https://www.genome.gov/about-genomics/teaching-tools/dna-origami)
- Genetics and Heredity (expression, mutations, and cancer)
- MSTP student-led activity

The general layout of day three included:

- Icebreaker
- Traits Bingo
- The Sun and You, UV damage lecture and lab activity (Carolina BioKits, item # 173608, DNA Damage: Studying the Impact of UV Light)
- Microscopic evaluation of normal and cancerous tissues with UAB pathologists
- Review Kahoot

The general layout of day four included:

- Icebreaker
- Cancer Blood detection lecture and activity (Edvotek, item # 141, Blood-based Cancer Diagnostics)
- Mutation telephone game
- Yeast plate results review
- Genetics Counselor
- Cancer Biology Jeopardy

The general layout of day five included:

- Pre-campus tour orientation
- Campus tour
- Scavenger hunt
- Graduate Student interactions
- Post-test and evaluation
- Camper certificates of completion and team prizes awarded

| **Supplementary Table 1.** *Pre- and Post-Test questions* |
| --- |
| **1. Human body cells have _____ pairs of chromosomes.** |
| a. 2 |
| b. 16 |
| c. 23 |
| d. 46 |
| **2.**  **Cancer is the breakdown of what normal body processes?** |
| a. Cell division |
| b. Cellular perspiration |
| c. External respiration |
| d. Internal respiration |
| **3. What makes an area qualify as medically underserved?** |
| a. Having too few primary care doctors in their region |
| b. Having a high rate of poverty in their region |
| c. Having a high rate of infant mortality in their region |
| d. Having no easily accessible hospitals in their region |
| e. All of the above |
| **4. How many nuclei can cancer cells have?** |
| a. Five |
| b. Two or more |
| c. None |
| d. None of the above |
| **5. Which of the following organs can develop cancer?** |
| a. Breast |
| b. Skin |
| c. Lung |
| d. Blood |
| e. All of the above |
| **6. Which type of cancer has the highest mortality rate (is the deadliest in the United States) in both men and women?** |
| a. Lung |
| b. Leukemia |
| c. Brain tumors |
| d. Liver |
| **7. Which of the following are types of cancer treatment?** |
| a. Chemotherapy |
| b. Radiation Therapy |
| c. Immunotherapy |
| d. All of the above |
| **8. All cells reproduce through cell division.** |
| a. True |
| b. False |
| **9. A tumor cell was initially found in the lungs. By week 10, tumor cells could be seen throughout other parts of the body. What characteristic allows cancer to spread?** |
| a. Metastasis |
| b. Benign |
| c. Metamorphosis |
| d. Hitch hikes a transport protein |
| **10. Which of the following best describes cancer as a disease?** |
| a. Transmitted by insects |
| b. Caused by infected water |
| c. Uncontrolled cell growth |
| d. Treated with antibiotics |
| e. Transmitted through the air |
| **11. What type of cancer treatment uses chemicals to target cells that divide rapidly?** |
| a. Surgery |
| b. Chemotherapy |
| c. Radiation |
| **12.** **Why do patients undergoing chemotherapy lose their hair?** |
| a. Chemotherapy targets rapidly dividing cells |
| b. Tis only a wig |
| c. Their barber showed up |
| d. The patients rip out their hair from stress |
| **13. Which of the following is a way to reduce your risk of getting cancer?** |
| a. Don’t smoke or use tobacco |
| b. Exercise |
| c. Use sunscreen to protect your skin while outside |
| d. Eat fruits and vegetables |
| e. All of the above |
| **14. Which of the following accurately describes the order of mitosis?** |
| a. Telophase, anaphase, metaphase, and prophase |
| b. Metaphase, prophase, telophase, and anaphase |
| c. Prophase, metaphase, anaphase, and telophase |
| d. Prophase, anaphase, metaphase, and telophase |
| **15. Tumor repressor genes are responsible for inhibiting cell growth?** |
| a. True |
| b. False |
| **16. Which of the following statements about mitosis/meiosis is correct?** |
| a. Mitosis results in four identical daughter cells |
| b. Meiosis results in two identical daughter cells |
| c. Mitosis results in two identical daughter cells |
| d. Meiosis functions for growth and repair |
| **17. Which of the following represents a DNA start codon?** |
| a. ATC |
| b. TAG |
| c. AAG |
| d. ATG |
| **18. What are the four bases that make up the code for DNA?** |
| a. T, A, G, and C |
| b. T, A, D, and F |
| c. T, E, C, and H |
| d. T, A, G, and E |
| **19. Which of the following accurately describes the idea of the central dogma?** |
| a. Protein → mRNA → DNA |
| b. DNA → mRNA → Protein |
| c. DNA → Protein → mRNA |
| d. None of the above |
| **20. What nitrogenous base is only found in RNA?** |
| a. uracil |
| b. thymine |
| c. guanine |
| d. cytosine |

|  | **Supplementary Table 2.** *BioBridge Student Quotes from Summers 2021-2023* |
| --- | --- |
|  | **Selected Quotes** |
| **Students liked BioBridge experiments and wanted more** | “Hands-on experiments - I got to see firsthand how things work and why and how they affect them.” “The micro-pipetting activity and blood marker experiment”  “When the facilitators explained positive and negative controls. Mainly because that explanation showed the importance of statistics in medical science.” “I really enjoyed the experiments with the yeast. The reason is that I am a visual learner and with us using yeast, I was able to actually see what we were learning about.” “The dna study because I learned so much about it and myself.” “The experiments were fun and you can apply the things you learned.” “My favorite part was the fun experiments we did even though we were learning at the same time.” |
| **Students shared cancer knowledge with their family and friends** | “On the way back home, I explained concepts we learned in camp, such as mitosis and how it is a type of cell division that results in two daughter cells that have the same type, kind and number of chromosomes as the parent cell. I also spoke about how cancer is uncontrolled cell division.” “I talked to my mom about how UV radiation affects you, and she told me how she had removed a benign spot on her body because it had the potential to be cancerous. Her grandmother had the same spot and it was cancer.” “I had conversations with my mother about what cancer is, how it can be dangerous, what they can do to help prevent themselves for developing cancer, and how to know if you have cancer.” “I talked with my dad about how important sunscreen was, especially since he’s had a malignant melanoma spot that was treated with surgery.” “[My parents and I] would talk about cancer, what it is, treatment, genetics, and many other topics.” “I taught [my grandmother] about the different things in cancer biology we are learning in this camp.” |
| **BioBridge stimulated career interest** | “I really liked the experiments and I liked when we got to talk to the professionals.”  “I had a conversation with Amber Jones about how she got past rough parts and why she chose what she does and it helped me make up my mind on what I want to pursue.” “I really liked learning about cancer research, it gave me a basic idea of how it could be done.” “I had conversations with my parents about the lessons of each day, and I discussed student life and careers with the speakers and facilitators.” “Guest speakers- we see firsthand people who have gone through programs and get valuable advice.” “Genetic Counseling was very interesting and thought-inducing.” |

**Supplementary Figure 1.** Results from individual questions on the pre-and post-assessment. The average student responses to individual items on the 20-question assessment for years 2 and 3 are shown. Data represent means ± s.e.m., n = 68. Students absent on the first or last day of camp were not included in the data set.  * p < 0.05 compared to pre-testing. Statistical analysis was performed using Student’s t-test (paired analysis).
